# A novel porous egg-white (EW)/titania composite photocatalytic material for efficient photodegradation applications

**DOI:** 10.1039/d0ra00730g

**Published:** 2020-02-27

**Authors:** Guo Feng, Feng Jiang, Zi Hu, Weihui Jiang, Jianmin Liu, Quan Zhang, Qian Wu, Qing Hu, Lifeng Miao, Si Cheng

**Affiliations:** National Engineering Research Center for Domestic & Building Ceramics, Jingdezhen Ceramic Institute Jingdezhen 333000 China fengguo@jci.edu.cn jiangweihui@jci.edu.cn +86 798 8499328 +86 798 8499328; Department of Material Science and Engineering, Jingdezhen Ceramic Institute Jingdezhen 333000 China jiangfeng@jci.edu.cn; Jiangxi Ceramic Research Institute Jingdezhen 333000 China

## Abstract

A novel porous egg-white (EW)/titania composite material was prepared *via* a facile nonaqueous precipitation method with EW as the porous skeleton. In a typical process, tetrabutyl titanate, a titanium precursor, was dissolved in ethanol to undergo a non-hydrolytic reaction with the aid of anhydrous formic acid under ultrasonication and form a porous structure with EW. The composite material was characterized by BET, XRD, FTIR spectroscopy, TEM, FE-SEM and photocatalytic degradation test. The results show that formic acid changes the characteristic structure of tetrabutyl titanate, increases the polarity of its C–O and Ti–O bonds, and promotes the non-hydrolytic de-etherization poly-condensation reaction. After ultrasonic treatment, the reaction product was rearranged to form anatase titania on EW to form a porous structure. The porous composite material had a mean pore size of 15.8 nm, BET surface area of 325.5 m^2^ g^−1^ and exhibited an excellent photocatalytic activity. The degradation rate of methyl orange using the EW/titania composite material reached 99.9% in 50 minutes, exhibiting an attractive prospect in wastewater treatment.

## Abstract 2

A novel porous egg-white (EW)/titania composite material was prepared *via* a facile nonaqueous precipitation method with EW as the porous skeleton.

## Graphical abstract

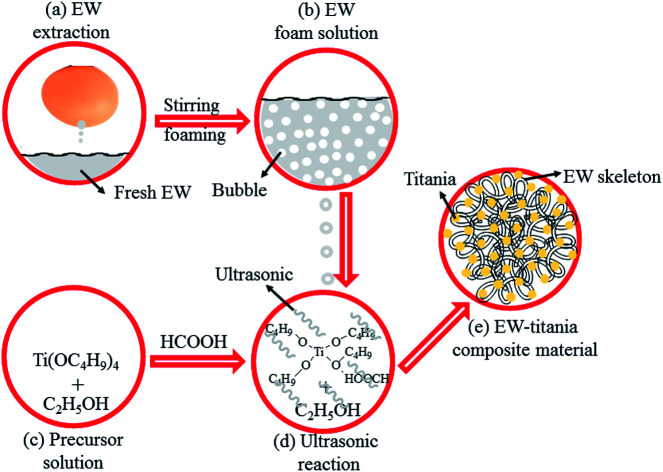

## 1 Introduction

Titania, a semiconductor with an energy gap (*E*_g_) of 3.2 eV, has captured much attention as an efficient photocatalyst for its promising photocatalytic activity, excellent chemical stability, superior oxidation capability, low cost and nontoxicity.^1,2^ It is universally acknowledged that the photocatalytic activity of titania strongly depends on its specific surface area, crystallinity, morphology and crystal facets.^3–6^ In most cases, titania preparation needs thermal treatment to get high crystallinity in final products. However, thermal treatment leads to an increase in the grain size and decrease in the specific surface area of the products. In order to alleviate these problems, much attention has been focused on using different second-phase heterogeneous porous materials such as titania carriers to reap the benefits on improving the specific surface area and photocatalytic performance of the materials.^7,8^ Unfortunately, the thermal treatment also limits the selection range of these porous carriers.

In the past several decades, there have been many attempts to develop a titania preparation process that uses thermal treatment, such as the hydrothermal method^9,10^ and nonhydrolytic sol–gel method.^11,12^ These methods inevitably require harsh high-pressure environment or need titanium tetrachloride to react with reactive oxygen species, *i.e.* tertbutyl alcohol^11^ or benzyl alcohol,^12^ with the help of ultraviolet radiation. Previously, we developed a novel non-aqueous precipitation method for titania preparation without thermal treatment.^13^ Herein, egg-white (EW) was employed to provide a porous skeleton for titania preparation to form a novel porous EW/titania composite photocatalytic material.

## 2 Experiments

### 2.1 Sample preparation

In a typical process, egg-white was extracted using a simultaneous purging-extraction apparatus. All other reagents were of reagent grade and used without further purification. 10 mL tetrabutyl titanate (Ti(OBu)_4_) was dissolved in 100 mL anhydrous ethanol under continuous stirring. As for the ET^#^ sample, 4.5 mL anhydrous formic acid (HCOOH) was added to the above solution.

**Fig. 1:**
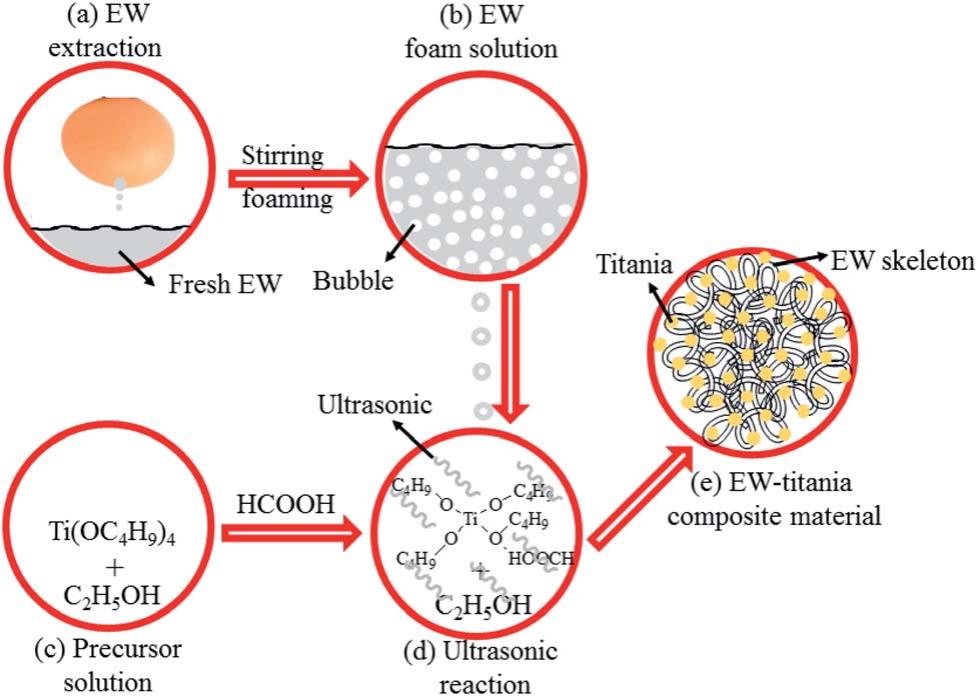
Schematic illustration of the preparation process for porous EW/titania composite material.

Subsequently, 1.932 g fresh egg-white was stirred severely for 3 min to make it foamy and added dropwise to the reaction system with continuous and vigorous stirring. The samples were ultrasonicated for 15 min after the fresh egg-white foam liquid was completely added to the reaction system. White and cotton-like precipitate was then separated, which was washed with ethanol twice. The precipitate was dried at 60 °C for 6 h to get the final product. Fig. 1 schematically illustrates the process for EW/titania composite material preparation.

### 2.2 Characterization

The crystalline phase of the samples prepared without HCOOH (W^#^) and with HCOOH (ET^#^) was characterized by X-ray diffractometry (XRD, D8 Advance, Bruker, Germany) at 40 kV and 30 mA using CuKα radiation. The morphology and microstructure of the ET^#^ sample was determined by transmission electron microscopy (TEM, SU-8010, JEOL, Japan) equipped with Image J software. Morphology of the ET^#^ sample was also characterized by field-emission scanning electron microscopy (FE-SEM, JSM-6700E, JEOL, Japan) equipped with Energy Dispersive Spectroscopy (EDS). The BET (Brunauer–Emmett–Teller) specific surface area (SBET) was measured by BET isothermal nitrogen adsorption using Micromeritics surface area analyzer (BRT, ASAP2020M, Micromeritics, America). Fourier transform infrared tests were characterized by an infrared spectrometer (FT-IR, Nicolet 5700, Thermo Electron Scientific Instruments Corp., America).

Methyl orange (C_14_H_14_N_3_NaO_3_S, MO) degradation was tested in aqueous solution using the EW/titania composite material at room temperature. Specifically, 0.1 g EW/titania material was added to 50 mL MO solution (10 mg L^−1^) with ultraviolet irradiation (30 W UV lamp with wavelength of 254 nm). The EW/titania material suspension was centrifuged and the concentration of the centrifuged solution was monitored by an ultraviolet-visible spectrophotometer (UV-Vis, Lambda 850, PerkinElmer Instrument Company, America). Corresponding comparison tests were also performed for P_25_ nanoparticles (Degussa).

## 3 Results and discussion

**Fig. 2:**
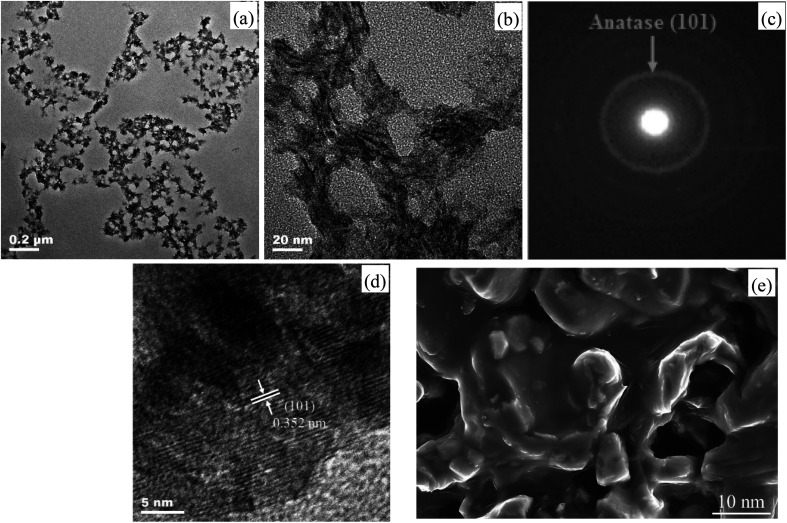
(a) and (b) TEM, (c) SAED, (d) HRTEM and (e) FE-SEM images of the as-prepared ET^#^ EW/titania material.

TEM images (a) low magnification and (b) high magnification, (c) SAED pattern, (d) HRTEM image and (e) FE-SEM graph of the as-prepared ET^#^ EW/titania material are respectively shown in Fig. 2.

Fig. 2(a) and (b) give direct evidence on porous architectures for EW/titania material displaying high porosity. A mutually connected network of random-sized pores with the sizes in the range from about 3 nm to 0.1 μm. With the aid of Image J software installed on the TEM provided by JEOL, the statistic mean pore size was calculated to be about 15.5 nm. Fig. 2(c) and (d) reveal the highly crystalline nature of the EW/titania material. The ring diffraction patterns in Fig. 2(c) are suggestive of the polycrystalline anatase nature of titania nanostructures. Concurrently, the HRTEM graph of the sample in Fig. 2(d) indicates clear lattice fringes with a regular interval of 0.352 nm, which corresponds to the interplanar spacing of (101) anatase planes. Nevertheless, the as-prepared titania particles have an irregular shape, which may be due to the coordination between the titanium precursor and C=O, N–H, O–H groups present in proteins of EW.^14^ The FE-SEM graph shown in Fig. 2(e) also demonstrates the co-existence of nano-titania particle, EW skeleton and nano-pore in the EW/titania composite material. Moreover, buckling and rough structure of EW protein is also clearly shown in the FE-SEM graph. Furthermore, it is interesting to note that nano-titania particles have been embedded into EW protein, ensuring the role of EW protein as an excellent skeleton.

Table 1 depicts the pore size distribution, mean pore size, and BET surface area of the as-prepared ET^#^ EW/titania material. The pore size distribution of the as-prepared ET^#^ EW/titania material is narrow, in the range of 2.2–105.4 nm, and the mean pore size of the sample is 15.8 nm. The BET surface area of this material is 325.5 m^2^ g^−1^, which is much higher than that of general single titania material.^15,16^

**Table 1:** Pore size distribution, mean pore size, BET surface area of ET^#^ EW/titania material

Indexes	Pore size distribution (nm)	Mean pore size (nm)	BET surface area (m^2^ g^−1^)
Values	2.2–105.4	15.8	325.5

**Fig. 3:**
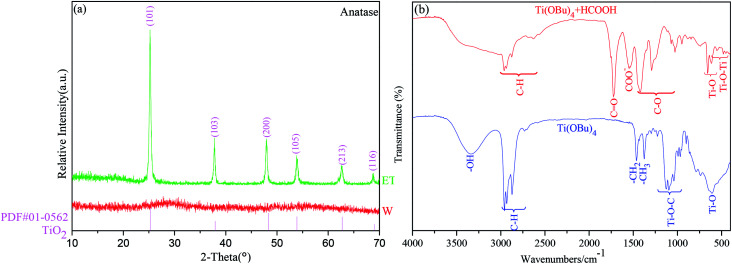
(a) XRD patterns and (b) FT-IR spectra of samples.

The XRD patterns of the samples without HCOOH (W^#^) and with HCOOH (ET^#^) are presented in Fig. 3(a). The XRD pattern of W^#^ has no diffraction peak, which indicates that it is amorphous. In sharp contrast, the synthesized ET^#^ sample is indexed to the single anatase titania (PDF card no. 01-0562) phase, which corresponds to the *I*4_1_/*amd* (141) space group, and its unit cell volume is 130.4 Å^3^. No impurity phases such as brookite or rutile phase of TiO_2_ or any other phase are indexed in the sample. In addition, the crystalline TiO_2_ percentage is, as calculated by Jade software, 85.8%, which is much higher than that of our previous sample (46.2%) prepared using LiI as the catalyst^17^ under the same test and calculation conditions. Moreover, the mean crystallite size of anatase titania is found to be 10.6 nm by using the Scherrer's formula.

To investigate the mechanism of HCOOH, FT-IR tests were performed on samples of Ti(OBu)_4_ and mixture of Ti(OBu)_4_ and HCOOH. Fig. 3(b) shows the results of FT-IR spectroscopy tests. Compared with the spectrum of Ti(OBu)_4_, the infrared spectrum of the mixture of Ti(OBu)_4_ and HCOOH shows new bands at 1725 cm^−1^, 1548 cm^−1^, 1291 cm^−1^ and 1071 cm^−1^, which are related to the vibrations of COO. Whereas, the bands observed in the infrared spectrum of Ti(OBu)_4_ at 1125–1034 cm^−1^, ascribed to the vibrations of Ti–O–C, disappear. Ti–O–C is generally thought to be the characteristic bond in Ti(OBu)_4._ Its disappearance in the sample is suggestive that the addition of HCOOH has changed the structure of Ti(OBu)_4_. In addition, the bands at 611 cm^−1^ and 1028 cm^−1^ ascribed to Ti–O and C–O vibrations, exhibit obvious red shift to 614 cm^−1^ and 1046 cm^−1^, respectively. Meanwhile, it is also worth noting that the band of –OH in anhydrous formic acid also disappears in the spectrum of the mixture. These results clearly prove that formic acid has coordinated with Ti(OBu)_4_, and the process of chemical reaction is presented in formula (1). H atom of HCOOH shown at position 1 coordinates with the O atom at position 2 of Ti(OBu)_4_ to forma hydrogen bond. Electron-withdrawing inductive effect of H atom greatly reduces the charge density on O in Ti(OBu)_4_ and weakens the bond strength of the C–O bond and the Ti–O bond. It also increases the polarity of Ti–O and C–O bond, making it easy for them to be nucleophilically attacked and result in nonhydrolytic deetherization polycondensation. This is also the fundamental cause for the formation of anatase titania in the ET^#^ sample with HCOOH, whereas it cannot form anatase titania in the sample W^#^.1
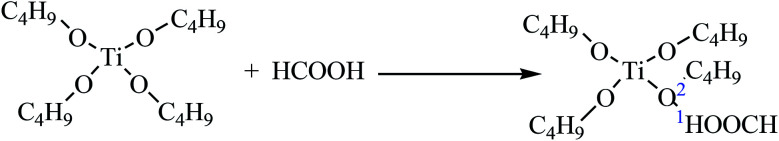


**Fig. 4:**
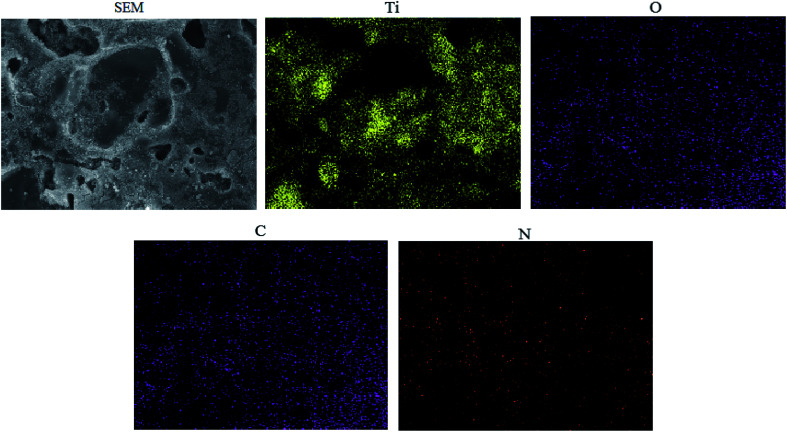
EDS elemental mapping of ET^#^ EW/titania composite.

EDS elemental mapping of ET^#^ EW/titania composite is presented in Fig. 4. The presence and uniform distribution of N indicates that the egg white and TiO_2_ have effectively achieved a homogeneously distributed composite.

**Fig. 5:**
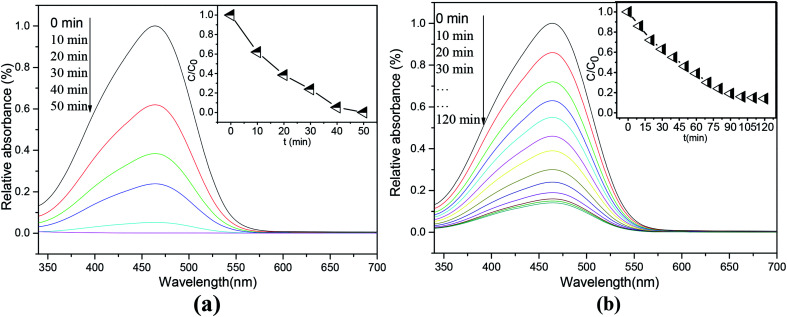
Absorption spectra of MO solutions after photodegradation tests at different time intervals. (a) ET^#^ EW/titania composite (b) P_25_.

The photocatalytic activity of the as-prepared ET^#^ EW/titania composite material was studied by measuring its photodegradation performance on methyl orange under UV light. The results are shown in Fig. 5(a). As a comparison, Fig. 5(b) also presents corresponding photodegradation results of P_25_ nanoparticles (Degussa). Characteristic absorption of MO at 464 nm is selected to detect its concentration. ET^#^ EW/titania composite material shows a high photocatalytic activity. It removes about 99.9% of MO in 50 min, the photodegradation performance, which is much higher than that of relative reports.^17,18^ As a comparison, with the same test conditions, P_25_ nanoparticles (Degussa) remove only 54.1% of MO in 50 min. In addition, as the development trend of the degradation results of P_25_, it can be inferred that much longer time than 50 min is needed for P_25_ to remove 99.9% of MO.

The attractive photocatalytic activity of EW/titania composite material may be explained by the following reasons: (1) high BET surface area with high nano-porous structure; (2) the binary heterostructure of EW/titania restrains electron–hole recombination centers and facilitates the conduction of photoelectron–hole pair; (3) EW protein is conducive to the adsorption of organic pollutants such as MO.

## 4 Conclusion

A novel porous EW/titania composite photocatalytic material has been developed in the present work *via* a simple and green method. Formic acid changes the characteristic structure of tetrabutyl titanate and increases the polarity of its Ti–O and C–O bonds. It also facilitates the de-etherization polycondensation reaction of tetrabutyl titanate. With the aid of ultrasonication and EW, a novel porous EW/titania composite photocatalytic material was produced. Concurrently, the EW/titania composite material is highly porous with a high BET surface area of 325.5 m^2^ g^−1^. Hence, this novel porous EW/titania composite material shows an excellent photocatalytic performance. The process for the preparation of the EW/titania composite photocatalytic material is facile, inexpensive, and highly efficient.

## Conflicts of interest

There are no conflicts to declare.

## Supplementary Material

## References

[cit1] Valden M., Lai X. (2004). Science.

[cit2] Ohno T., Tsubota T., Toyofuku M., Inaba R. (2004). Catal. Lett..

[cit3] Wu N., Wang J., Tafen D. N., Wang H., Zheng J. G., Lewis J. P. (2010). J. Am. Chem. Soc..

[cit4] Pietro G., Giovanni M., Carmen G. (2016). Process. Appl. Ceram..

[cit5] Wang Y., Zhang H. M., Han Y. H., Liu P. R., Yao X. D., Zhao H. J. (2011). Chem. Commun..

[cit6] Du Y. E., Niu X. J., Li W. X., An J., Liu Y. F., Chen Y. Q., Wang P. F., Yang X. J., Feng Q. (2019). Materials.

[cit7] Marija M., Ljubica M. N. (2014). Process. Appl. Ceram..

[cit8] Erno E. K., Goran C. B. (2016). Process. Appl. Ceram..

[cit9] Man Y., Zhao J., Lv S., Lu K. (2017). Ceram. Int..

[cit10] Hua G. Y., Cheng H. S., Shi Z. Q., Jin Z., Gang L., Smith S. C. (2008). Nature.

[cit11] Zhu J., Bian Z. F., Ren J., Liu Y. M., Cao Y., Li H. X. (2007). Catal. Commun..

[cit12] Wang C. C., Ying J. Y. (1999). Chem. Mater..

[cit13] Feng G. , Xu G. R., Jiang W. H., Liu J. M., Zhang Q., Wu Q. and Miao L. F., *Chin. Pat.*, ZL 201610804064.6, 2018

[cit14] Yin L. Y., Zhou X. G., Yu J. S., Wang H., Zhe L. (2014). Ceram. Int..

[cit15] Xu G., Zheng Z., Wu Y., Na F. (2009). Ceram. Int..

[cit16] Wang Z. M., Li Y. M., Shen Z. Y., Hong Y., Zuo J. L. (2011). J. Ceram..

[cit17] Liao Q. L., Jiang W. H., Feng G., Peng Y. F. (2014). J. Ceram..

[cit18] Li Y. F., Xu D., Oh J. I., Shen W., Xi L., Ying Y. (2012). ACS Catal..

